# A study on the relationship between teacher feedback types, self-efficacy, and intrinsic motivation among violin learners

**DOI:** 10.3389/fpsyg.2026.1985014

**Published:** 2026-09-30

**Authors:** Xiaolong Ye, Jingzhi Yang, Qi Song

**Affiliations:** 1 School of Music and Dance, Quanzhou Normal University, Quanzhou, Fujian, China; 2 Music College, Capital Normal University, Beijing, China

**Keywords:** adult violin learning, intrinsic motivation, learning stage, moderated mediation, self-efficacy, teacher feedback

## Abstract

**Introduction:**

To explore the psychological mechanisms of persistence in adult instrumental learning, this study investigates the associations among different types of teacher feedback, self-efficacy, and intrinsic motivation, and examines the moderating role of learning stages.

**Methods:**

Using a cross-sectional survey design, scale and open-ended text data were collected from 743 adult violin learners. A moderated mediation model was tested through multiple regression and the Bootstrap method, supplemented by thematic analysis to provide a qualitative context.

**Results:**

Data analysis indicates: (1) Positive feedback and constructive feedback are significantly and positively associated with intrinsic motivation (*β* = 0.207, *p* < 0.001; *β* = 0.342, *p* < 0.001), while negative feedback shows a significant negative association (*β* = −0.318, *p* < 0.001); (2) Self-efficacy demonstrates a significant partial mediating association between the three types of feedback and intrinsic motivation, with indirect effect estimates of 0.103, 0.098, and −0.097, respectively (95% CIs all exclude 0); (3) The learning stage significantly moderates the conditional indirect effects: as the learning stage advances, the indirect association of positive feedback on intrinsic motivation via self-efficacy gradually weakens (index of moderated mediation = −0.045), whereas the indirect association of constructive feedback gradually strengthens (index of moderated mediation = 0.038). Open-ended texts echo these statistical paths at the micro-level.

**Discussion:**

The study shows that there are heterogeneous and dynamic statistical connections between teacher feedback and adult intrinsic motivation, and self-efficacy is a key pathway to understanding this connection. In practice, the provision of instructional feedback content should match the learners’ experience stage.

## 1 Introduction

### 1.1 Research background

Adult violin learning is characterized by multiple technical requirements, long practice cycles, and relatively slow progress feedback. Skills such as posture, bowing, intonation, rhythm, shifting, and musical expression need to be gradually established through sustained practice; short-term investment may not necessarily translate into progress that learners can directly perceive. Repetitive practice may also be accompanied by movement adjustments, error corrections, and periodic stagnation, leading to experiences of boredom, frustration, or a sense of inadequacy (Ampler et al., 2024; Macnamara et al., 2014; Nash, 2025).

Adult learners typically need to schedule practice around work, family, and personal life, making time allocation and learning continuity susceptible to practical constraints (Jiang et al., 2026). Compared to learners aiming for school admission, grading exams, or professional development, the internal and external constraints for adults participating in violin learning (whether for interest, aesthetic experience, personal development, or career advancement) are unique (Creech et al., 2013). Under these circumstances, whether learners are willing to practice proactively, whether they can derive interest from learning activities, and whether they remain willing to engage when facing difficulties become critical issues for sustaining learning participation (Ruth and Müllensiefen, 2021).

Intrinsic motivation is an individual’s tendency to participate in an activity based on the interest, challenge, or satisfaction derived from the activity itself. While adult learners may begin playing the violin for various background reasons—such as initial aesthetic interest, personal growth, or external social influences—sustaining this demanding practice relies heavily on their ongoing psychological state. Therefore, it is necessary to focus on their intrinsic motivation, a dynamic empirical construct distinct from those original starting motives. For adult violin learners, intrinsic motivation is manifested not only in their interest in musical activities but also in their autonomy in scheduling practice, taking the initiative to deal with technical issues, and their willingness to stay engaged even in the absence of external rewards (Wieser et al., 2024). Since the formation of violin skills takes a long time, intrinsic motivation is closely linked to learners’ practice engagement and intention to continue learning. Exploring factors related to intrinsic motivation helps in understanding the psychological experiences of adult learners in long-term instrumental training (Földi et al., 2024).

Classroom interaction constitutes an important part of the adult violin learning environment. Teachers not only provide technical demonstrations and practice tasks, but also convey feedback to learners through verbal evaluation, problem diagnosis, and emotional expression, with the digital age even expanding feedback into online formats (Zhen and Yahaya, 2026). Feedback may involve performance outcomes, learning effort, technical issues, and directions for subsequent practice; the information content and expression methods contained in different feedback vary. Learners also interpret feedback based on their own experience, current proficiency level, and the classroom context (Hattie and Timperley, 2007).

This study distinguishes teacher feedback into positive feedback, constructive feedback, and negative feedback. Positive feedback mainly refers to the teacher’s affirmation of the learner’s effort, progress, or performance; constructive feedback emphasizes problem identification, improvement methods, and actionable practice suggestions; negative feedback includes denial, blame, derogatory comparisons, or criticism lacking improvement information. These three types of feedback may correspond to different ability judgments and classroom perceptions, and may also exhibit different directional associations with learners’ intrinsic motivation (Brandmo and Gamlem, 2025).

### 1.2 Research questions

Existing research on teacher feedback has largely focused on academic achievement, skill performance, and error correction, leaving further room for discussion on the relationship between feedback and learners’ psychological experiences (Gill et al., 2026). For adult violin learners at different stages, feedback is not merely technical information, but may also serve as a basis for judging their own learning abilities, understanding learning difficulties, and evaluating classroom relationships (Fancourt et al., 2021). Therefore, solely examining the direct association between teacher feedback and intrinsic motivation makes it difficult to explain the potential psychological connections between them.

Self-efficacy provides a theoretical perspective for understanding this issue. Self-efficacy is an individual’s judgment of their capability to complete a specific task or cope with related difficulties. In the context of violin learning, it can manifest as the learner’s confidence in mastering playing skills, correcting errors, completing practice tasks, and dealing with learning bottlenecks (Bi and Rauduvaitė, 2025). Teacher feedback, self-efficacy, and intrinsic motivation may form a statistical association path: teacher feedback is related to learners’ ability judgments, which in turn correspond to their learning interest and autonomous engagement. Incorporating self-efficacy into the model allows for testing whether there is an indirect association between teacher feedback and intrinsic motivation (Miao et al., 2025).

The learning stage may also constitute a boundary condition for the above relationships. Beginners are still in the stage of establishing basic movements, intonation, and rhythmic rules, and their judgment of their own performance may rely more on teacher evaluations. Advanced learners have accumulated certain playing experience, and the problems they face gradually shift to technical details, musical processing, and practice strategies; their focus on feedback content may change accordingly (Parncutt and McPherson, 2002). Whether different learning stages correspond to different feedback association patterns needs to be tested empirically.

Existing studies also rely heavily on closed-ended scales. Scale data are suitable for presenting overall associations between variables, but it is difficult to explain how learners understand specific feedback and in what forms such feedback appears in the classroom. Open-ended responses can preserve the context in which feedback occurs, presenting learners’ descriptions of teachers’ language, technical guidance, and emotional expressions. Combining scale data with open-ended materials can provide contextual supplementation for statistical relationships.

### 1.3 Research objectives

Focusing on adult violin learners, this study adopts a cross-sectional questionnaire survey design to investigate the relationships among teacher feedback, self-efficacy, intrinsic motivation, and learning stages. The research objectives include: testing the statistical associations of positive feedback, constructive feedback, and negative feedback with intrinsic motivation; examining the indirect relationship of self-efficacy between the three types of teacher feedback and intrinsic motivation; analyzing whether the learning stage moderates the association between teacher feedback and self-efficacy; and further testing whether conditional indirect effects differ across learning stages.

The study also incorporates an open-ended question to conduct a thematic analysis of the feedback experiences described by learners, serving to supplement the relational models presented by the scale results. Additionally, unlike previous studies that mostly focused on single-level groups, the sample in this study covers a broad range of adult learners from beginners to professional levels, which enables this study to effectively test the dynamic moderating role of the “learning stage” in teacher feedback and psychological mechanisms.

This study builds upon Social Cognitive Theory (SCT) and Self-Determination Theory (SDT). SCT is used to explain the relationship between learning environments, self-efficacy, and learning behaviors (Bandura, 1997), while SDT is applied to understand the connection between the needs for competence, relatedness, autonomy, and intrinsic motivation (Ryan and Deci, 2020). The two theoretical perspectives jointly form the analytical framework for teacher feedback, self-efficacy, and intrinsic motivation. Rather than treating these theories in isolation, the study integrates them to examine how learners’ confidence in tackling specific technical tasks—rooted in self-efficacy—serves as a crucial pathway to fulfilling their broader psychological need for competence. This integrated perspective provides a more nuanced lens to understand how teacher feedback translates into sustained practice.

## 2 Theoretical background and hypotheses

### 2.1 Social cognitive theory and self-efficacy

Social Cognitive Theory posits that there are interconnections among individuals, environments, and behaviors, and that learners form judgments about their own abilities based on personal experiences, task performances, and social information (Bandura, 1986). Self-efficacy is one of the core concepts of this theory, referring to an individual’s belief in their ability to complete a specific task or cope with related difficulties. It possesses task and context-specific features, rather than being a generalized evaluation of personal ability (Bandura, 1997).

Violin learning involves continuous skill practice and performance evaluation. Learners need to judge whether they can complete technical movements, understand teacher requirements, correct playing issues, and achieve staged goals. Teacher feedback is one of the informational sources for this judgment, but learners do not interpret all feedback in the same way. Whether the feedback content is specific, whether it points to adjustable behaviors, and whether it includes ability evaluations, may all exhibit different associations with self-efficacy (Bi and Rauduvaitė, 2025; He et al., 2025).

Positive feedback typically includes affirmation of effort, progress, or performance, which can provide learners with ability confirmation. Constructive feedback focuses on problem explanation, technical demonstration, and practice suggestions, providing task information and directions for improvement. Negative feedback may include denial, blame, or comparison; its relationship with learners’ ability judgments depends on the feedback content and how it is interpreted. Therefore, it is necessary to differentiate the three types of feedback and separately examine their relationships with self-efficacy and intrinsic motivation (Brandmo and Gamlem, 2025).

### 2.2 Self-determination theory and intrinsic motivation

Self-Determination Theory views autonomy, competence, and relatedness needs as crucial dimensions for understanding learning motivation (Ryan and Deci, 2020). Autonomy reflects an individual’s sense of choice and self-directed participation in learning activities; competence involves the individual’s experience of completing tasks and improving abilities; relatedness refers to the individual’s desire to gain understanding, respect, and support in social interactions.

Adult violin learning has strong autonomous participation characteristics (Evans, 2015). Whether learners are interested in practice activities, whether they can feel capability development, and whether they are respected in the classroom may all be related to their intrinsic motivation. Teacher feedback contains both task information and relational information. Affirmation of effort and progress may be related to experiences of competence and relatedness; specific technical suggestions may be related to a sense of task mastery; negative evaluations may co-occur with lower competence or poorer classroom relationship experiences (Howard et al., 2021).

Rather than functioning as isolated frameworks, Social Cognitive Theory focuses on task-specific ability judgments, while Self-Determination Theory emphasizes broader psychological needs and motivation; the two are deeply connected at the level of competence experience. Specifically, in feedback contexts, a learner’s domain-specific self-efficacy—their concrete belief in executing specific playing tasks—serves as the foundational pathway to satisfying the psychological need for competence. When constructive qualitative feedback helps learners feel confident in overcoming technical barriers, this specific efficacy directly nurtures their broader sense of competence, thereby fostering intrinsic motivation. Self-efficacy can thus serve as a rational indicator of ability judgments to examine the statistical mediation relationship between teacher feedback and intrinsic motivation (Zhang, 2025).

### 2.3 Teacher feedback and intrinsic motivation

Positive feedback conveys to learners that their efforts, progress, or performances are being noticed. The practice results of adult learners may not be directly perceived in the short term, and the teacher’s affirmation of staged changes may be related to the learners’ ability confirmation and classroom engagement. Consequently, learners with higher levels of positive feedback may simultaneously report higher intrinsic motivation (Öztürk et al., 2025).

Constructive feedback not only evaluates performance results but also includes problem identification, cause explanation, and practice suggestions. Such feedback provides learners with clearer directions for adjustment, allowing technical difficulties to be broken down into specific tasks. There may be a connection between constructive feedback and a sense of problem controllability and task mastery, thus it may also be positively associated with intrinsic motivation (Guo et al., 2025).

Negative feedback usually carries negative, blaming, or comparative information. If the feedback lacks specific directions for improvement, learners may find it difficult to translate it into practice tasks and may concurrently report lower competence perception and learning interest (Shriya and Bhuyan, 2025). Based on the above discussions, this study proposes:

*H1a*: Positive feedback is significantly and positively associated with the intrinsic motivation of adult violin learners.

*H1b*: Constructive feedback is significantly and positively associated with the intrinsic motivation of adult violin learners.

*H1c*: Negative feedback is significantly and negatively associated with the intrinsic motivation of adult violin learners.

### 2.4 The mediating role of self-efficacy

The relationship between teacher feedback and intrinsic motivation may not be solely direct. The competence, task, and relational information contained in feedback may be related to learners’ self-efficacy judgments, which in turn are linked to learning interest, task choice, and willingness to engage. Therefore, self-efficacy may present a statistical mediating relationship between teacher feedback and intrinsic motivation (Miao et al., 2025).

Positive feedback mainly provides performance confirmation, and its association with intrinsic motivation may partly manifest as an indirect relationship through self-efficacy. Constructive feedback provides problem diagnosis and improvement strategies, and learners’ judgments on task operability may also be related to self-efficacy. The negative information contained in negative feedback may co-occur with lower self-efficacy and further manifest as a negative indirect association with intrinsic motivation (Arrington, 2023; Gill et al., 2026). Accordingly, it is proposed:

*H2a*: Self-efficacy has a significant statistical mediating effect between positive feedback and intrinsic motivation.

*H2b*: Self-efficacy has a significant statistical mediating effect between constructive feedback and intrinsic motivation.

*H2c*: Self-efficacy has a significant statistical mediating effect between negative feedback and intrinsic motivation.

### 2.5 The moderating role of learning stage

The learning stage reflects learners’ prior experience, current technical tasks, and problem identification abilities. Beginners are still establishing basic playing movements and evaluation standards, and their judgments of their own performance may heavily reference teacher evaluations. The affirmative information contained in positive feedback may thus form a stronger association with beginners’ self-efficacy.

Advanced learners already possess a certain amount of playing experience, their judgments on technical issues are more specific, and their classroom needs may also shift toward problem diagnosis, movement adjustment, and practice strategies (Parncutt and McPherson, 2002). The task information provided by constructive feedback highly corresponds to their current learning needs. Therefore, the association between constructive feedback and self-efficacy may strengthen as the learning stage advances. Based on the above theoretical deductions, this study proposes:

*H3a*: The learning stage significantly moderates the relationship between positive feedback and self-efficacy; compared to intermediate and advanced learners, this positive relationship is stronger among lower-level learners.

*H3b*: The learning stage significantly moderates the relationship between constructive feedback and self-efficacy; compared to lower-level learners, this positive relationship is stronger among intermediate and advanced learners.

Regarding negative feedback, existing literature presents conflicting theoretical perspectives on whether its negative association with motivation attenuates as learning stage deepens (e.g., advanced learners developing higher psychological resilience; Creech et al., 2013) or intensifies (e.g., advanced learners possessing stricter self-evaluative standards; Bonneville-Roussy and Bouffard, 2015). Given these contrasting perspectives, this study does not predefine a conditional hypothesis for negative feedback. Instead, it is included in the model to evaluate its direct and mediated associations, leaving its dynamic evolution across stages as an open theoretical area for subsequent exploratory analysis.

### 2.6 Moderated mediation relationship

If the learning stage moderates the relationship between teacher feedback and self-efficacy, the indirect effect of teacher feedback on intrinsic motivation via self-efficacy may also change with the learning stage. This means that the strength of the indirect effect corresponding to the same type of feedback may not be fixed but rather related to the learner’s current stage.

Positive feedback and constructive feedback contain different informational focuses, and learners’ understanding of this information may also evolve with experience accumulation. The learning stage may thereby constitute a conditional variable in the statistical path of teacher feedback, self-efficacy, and intrinsic motivation (Wieser et al., 2024). To test this conditional indirect relationship, this study proposes:

*H4*: The learning stage significantly moderates the indirect effect of teacher feedback on intrinsic motivation via self-efficacy, and the strength of this indirect effect varies across different learning stages.

Synthesizing the deductive threads of Social Cognitive Theory (SCT) and Self-Determination Theory (SDT), the core conceptual framework and hypothesis matrix proposed in this study are shown in Figure 1.

**Figure 1:**
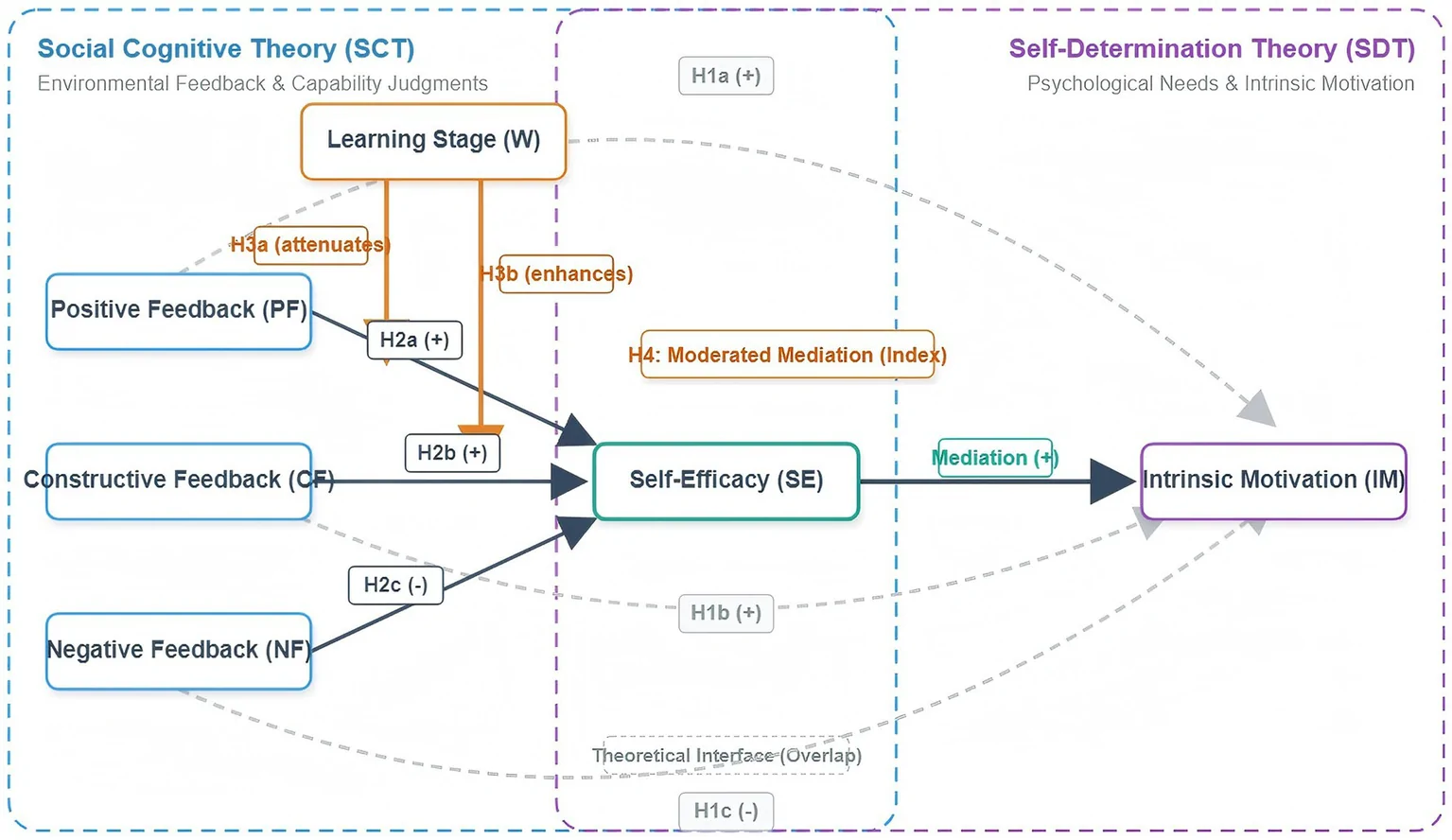
Conceptual framework and theoretical hypothesis paths integrating SCT and SDT. Solid lines represent mediation hypotheses, dashed lines represent direct association hypotheses; positive and negative theoretical expectations between variables are marked in parentheses; H4 represents the overall moderated mediation hypothesis.

## 3 Methodology

### 3.1 Research design

This study adopts a cross-sectional questionnaire survey design, taking adult violin learners as the research subjects to investigate the associations among perceived teacher feedback, self-efficacy, and intrinsic motivation, and to test the moderating effect of the learning stage. The questionnaire main body consists of three parts: demographic and learning background, psychological measurement scales, and an open-ended question. Given that the original versions of some core psychological scales used in this study were in foreign languages, the research team adopted a standard “Translation and Back-translation” procedure before finalizing the questionnaire, making appropriate contextual modifications and linguistic refinements according to the adult violin learning context to ensure cross-cultural semantic equivalence of the scales.

In the research model, positive feedback, constructive feedback, and negative feedback are independent variables, violin learning self-efficacy is the mediator variable, intrinsic motivation is the dependent variable, and the learning stage is the moderator variable; in addition, growth mindset and continuous learning intention are used as supplementary variables for auxiliary analysis. It must be specially declared that, since all data were collected at a single point in time, the results of this study are only used to reveal statistical correlations and structural associations among variables, and cannot be used to infer temporal sequences or strict causal relationships; the open-ended responses are used to present learners’ subjective descriptions of specific feedback experiences, providing rich contextual supplementation for the quantitative statistical results.

### 3.2 Participants and data collection

The survey subjects of this study are adult learners with violin learning experience. Data collection was mainly concentrated from March to June 2026. A total of 780 initial questionnaires were returned, and after rigorous data cleaning (such as removing samples with excessively short response times, regular response patterns, or severe missing data in key variables), 743 valid questionnaires were finally obtained, with a valid response rate of 95.3%.

The final sample achieved multidimensional and broad coverage. The questionnaire collected detailed demographic characteristics of the respondents (including age, gender, occupation or study status) as well as violin learning background (including cumulative learning duration, main teaching format, initial learning motivation, average daily practice duration, and time spent with the current teacher, etc.). During the structural model testing process, all these variables were included in the analysis as control variables to minimize potential interference of sample heterogeneity on the estimation of core variable relationships (complete classification definitions and data coding rules for each variable are detailed in Supplementary material Appendix B).

Furthermore, the core moderating variable “learning stage” in this study was operationalized by the respondents’ “self-rated violin level.” This variable is treated as an ordinal categorical variable in descriptive statistics to present the sample structure, while in subsequent moderation effect analyses, it is used as an interval moderating variable representing continuous progression in learning stages.

### 3.3 Measurement tools

All psychological scales used in this study were adapted from established instruments. Given the practical time constraints of adult learners, the final questionnaire deployed streamlined versions of these scales (retaining 3 to 4 highly representative items per construct) to ensure high response quality and mitigate fatigue. Except for basic information, all psychological scales use a 5-point Likert scale, with 1 representing “completely disagree” and 5 representing “completely agree.” The score for each construct is calculated by the arithmetic mean of its corresponding items; a higher score indicates a higher level of the trait reported by the learner. The psychological measurement items in the questionnaire are all positively scored, with no reverse-scored items. The complete Chinese and English scale items, scoring instructions, and Data Codebook used in this study are detailed in the Supplementary material Appendices A,B.

#### 3.3.1 Teacher feedback types

The teacher feedback type scale was developed based on the feedback framework by Hattie and Timperley (2007) and the study on perceived teacher feedback by Koka and Hein (2003), adapted to the context of violin teaching. The scale encompasses three dimensions: positive feedback, constructive feedback, and negative feedback, with each dimension containing 3specific items.

Positive feedback focuses on the teacher’s affirmation of learners’ performance, effort, and progress, such as “When I play well, the teacher explicitly praises me.” Constructive feedback emphasizes whether the teacher can point out specific problems and provide improvement methods, such as “When I make a mistake, the teacher not only points out the error but also explains exactly how to improve.” Negative feedback involves criticism lacking solutions, nitpicking, and negative information regarding ability or talent, such as “The teacher’s feedback sometimes makes me feel it’s because I lack talent, rather than an issue with practice methods.” Average scores are calculated for each of the three dimensions. A higher negative feedback score indicates more negative feedback experiences reported by the learner.

#### 3.3.2 Violin learning self-efficacy

The self-efficacy scale was adapted from the musical self-efficacy measurement tool by Ritchie and Williamon (2011) and contextually adjusted according to adult violin learning tasks. The scale contains 4 items covering learners’ capability judgments on mastering difficult phrases, correcting long-term technical issues, analyzing new pieces, and managing practice states. An example item is: “Even when encountering very difficult exercises or passages, I believe that as long as I practice scientifically, I can master them.” This scale mainly reflects learners’ capability beliefs in violin practice and technical tasks, differing from general confidence evaluations (Bandura, 1997).

#### 3.3.3 Intrinsic motivation

The intrinsic motivation scale for this study was adapted from the ‘Interest/Enjoyment’ subscale of the Intrinsic Motivation Inventory (IMI) and refers to the definition of intrinsic motivation by Ryan and Deci (2020). The scale contains 4 items measuring learners’ interest, enjoyment, engagement state, and autonomous practice tendency in violin practice. An example item is: “Even without teacher supervision or grading exam pressure, I will practice the violin on my own initiative.” A higher score indicates that learners participate in practice more based on the interest and satisfaction of the activity itself, rather than relying primarily on external requirements.

#### 3.3.4 Supplementary variables

The growth mindset scale was adapted from Dweck (2006), consisting of 3 items, primarily measuring whether learners believe playing ability can be improved through practice methods and continuous investment. The continuous learning intention scale references Bonneville-Roussy and Bouffard (2015), containing 2 items to understand learners’ willingness to continue learning in the coming year and to keep the violin as a long-term hobby. Both variables are mainly used for supplementary description and robustness analysis.

#### 3.3.5 Open-ended qualitative text collection

In addition to structured scales, an open-ended question (OEQ1) was placed at the end of the questionnaire as a qualitative data collection tool for the convergent mixed-methods design. This question asks respondents to describe a specific experience where teacher feedback made them feel extremely frustrated or motivated. The textual content from this part is not converted into quantitative scores but is extracted separately to illustrate the micro-psychological states underlying the quantitative hypotheses.

### 3.4 Data analysis strategies

Data analysis follows the basic workflow of “data preprocessing—measurement model testing—structural model testing—qualitative text analysis.” Quantitative data were processed using SPSS 27.0 (including the PROCESS macro) and AMOS 24.0, while qualitative texts were interpreted using Thematic Analysis (Braun and Clarke, 2006).

First, in the preprocessing and measurement model testing stage, the distributional shapes of the data were evaluated, and all continuous predictor variables were standardized (*Z*-score) prior to constructing interaction terms to mitigate multicollinearity. Subsequently, AMOS 24.0 was used to conduct a confirmatory factor analysis to test the reliability and validity of each construct, and Harman’s single-factor test was applied to assess the impact of common method bias (Podsakoff et al., 2003).

Second, in the structural model testing stage, multiple regression models were used to test the direct statistical associations of the three types of teacher feedback on intrinsic motivation; the SPSS PROCESS macro (Hayes, 2018) (based on 5,000 Bootstrap samples) was utilized to test the mediating effect of self-efficacy, the moderating effect of learning stages, and the conditional indirect effects. It is particularly noted that: First, to eliminate confounding effects among feedback types, when testing the effect of a single feedback type, the other two types of feedback and demographic background variables were statistically controlled as covariates. Second, the ordinal categorical indicator representing the “learning stage” was treated as an interval continuous variable in the moderation model. Although this processing assumes approximate linear intervals between adjacent stages (relevant methodological limitations will be addressed in the Discussion), in moderated mediation models involving complex interaction terms, treating multi-level ordinal categorical variables as continuous can effectively maximize the statistical power of the interaction effect tests, avoiding information loss caused by artificial dichotomization or multiple grouping, and thus more accurately capturing the dynamic trends of variable combinations as learning experience progresses. This is also a conventional practice for handling such data in current social science research. On this basis, considering that the real skill leap difficulty from ‘beginner’ to ‘professional’ is not strictly equidistant and non-linear inter-group spans may exist, in order to exclude latent algorithmic biases caused by the equidistant assumption on the moderation model, this study further introduced non-parametric tests (Kruskal-Wallis H test) and independent variable Dummy Coding strategies for statistical robustness analysis upon the completion of the main structural model testing, to cross-validate the reliability of the research conclusions. All statistical tests used *p* < 0.05 as the significance criterion.

Finally, the text data collected from the open-ended question were imported into NVivo 12 software for systematic qualitative coding. Following the principles of Thematic Analysis (Braun and Clarke, 2006), two independent coders evaluated the explicit feedback themes and implicit psychological statements within the narratives. This collaborative coding process achieved a high inter-coder reliability (Cohen’s *κ* = 0.85), with any minor discrepancies resolved through negotiated consensus. The resulting qualitative findings are interspersed after reporting the quantitative models, providing contextualized micro-supplements to the cross-sectional statistical relationships (all text quotes have been anonymized).

## 4 Quantitative and qualitative results

### 4.1 Sample basic information

This study finally included 743 violin learners; their demographic characteristics and specific learning backgrounds are detailed in Table 1. Overall, the sample broadly covers adult learning groups with different backgrounds.

**Table 1:** Demographic characteristics and learning background distribution of the sample (*N* = 743).

Variable	Category	*n*	Percentage
Age	18–25 years old	339	45.63%
	26–35 years old	250	33.65%
	36–45 years old	90	12.11%
	46–55 years old	49	6.59%
	56 years and older	15	2.02%
Gender	Male	238	32.03%
	Female	498	67.03%
	Other or prefer not to say	7	0.94%
Occupation or study status	Full-time non-music major student	256	34.45%
	Full-time music major student	34	4.58%
	Employed personnel	387	52.09%
	Freelancer, retired, or other	66	8.88%
Initial learning motivation	Interest in violin or music	306	41.18%
	Personal growth	251	33.78%
	Emotion regulation	111	14.94%
	External requirements	75	10.09%
Cumulative learning duration	1 year and under	316	42.53%
	1–3 years	256	34.45%
	3–5 years	110	14.80%
	Over 5 years	61	8.21%
Main teaching format	Offline one-on-one	415	55.85%
	Online one-on-one	184	24.76%
	Group or collective class	111	14.94%
	Mainly self-taught	33	4.44%
Average daily practice duration	Under 30 min	185	24.90%
	30–60 min	342	46.03%
	1–2 h	136	18.30%
	Over 2 h	80	10.77%
Time with current teacher	Under 6 months	415	55.85%
	6–12 months	201	27.05%
	1–3 years	84	11.31%
	Over 3 years	43	5.79%
Self-rated violin level	Beginner	313	42.13%
	Intermediate	226	30.42%
	Advanced	140	18.84%
	Professional	64	8.61%

Regarding demographic characteristics, respondents presented a typical profile dominated by young and middle-aged adults (18–35 years old accounting for nearly 80%), females, and employed individuals; their initial learning motivation mostly stemmed from an interest in music or a desire for personal growth (Note: “initial motivation” here is a categorical background variable and is fundamentally different in connotation from the “intrinsic motivation” measured by scales as a core empirical construct in this study). In terms of learning background, the sampled respondents were mostly in the beginning and intermediate stages of violin learning (low-to-intermediate levels and those learning for less than 3 years both accounted for over 70%), and highly relied on offline or online “one-on-one” teaching models. Moreover, the average daily practice time of most adults was limited to under 1 h, and the interaction cycle with their current teacher was relatively short (over 80% under 1 year).

### 4.2 Data quality and common method bias

The current data file contains 743 valid records. After checking the 22 psychological measurement items, no missing values, numbers out of the 1–5 scoring range, or records with identical responses across all scale items were found; no completely duplicated records were found after removing IDs.

Harman’s single-factor test showed that the first unrotated principal component explained 39.03% of the total variance, which is below the empirical threshold of 40% (Podsakoff et al., 2003), indicating no single factor explained the majority of the variance across items. Considering this proportion is close to the empirical threshold and Harman’s single-factor test cannot eliminate all common method bias, subsequent comprehensive judgments were combined with the multi-factor model fit results from the confirmatory factor analysis.

### 4.3 Reliability, convergent validity, and measurement model

The Cronbach’s *α* for positive feedback, constructive feedback, negative feedback, self-efficacy, and intrinsic motivation ranged from 0.826 to 0.877, and composite reliability (CR) ranged from 0.826 to 0.878. The standardized factor loadings of all items ranged from 0.755 to 0.865, and the average variance extracted (AVE) ranged from 0.613 to 0.676. The specific reliability and convergent validity indicators for each core construct are detailed in Table 2.

**Table 2:** Reliability and convergent validity of core constructs.

Construct	Number of items	Standardized loading range	Cronbach’s *α*	CR	AVE	Square root of AVE
Positive feedback (PF)	3	0.778—0.818	0.839	0.840	0.636	0.797
Constructive feedback (CF)	3	0.755—0.815	0.826	0.826	0.613	0.783
Negative feedback (NF)	3	0.794—0.865	0.860	0.862	0.676	0.822
Self-efficacy (SE)	4	0.746—0.831	0.877	0.878	0.644	0.802
Intrinsic motivation (IM)	4	0.779—0.814	0.869	0.869	0.624	0.790

The Cronbach’s *α* values for growth mindset and continuous learning intention are 0.822 and 0.833, respectively. The composite reliabilities of all core constructs are above 0.70, and AVEs are all above 0.50, meaning internal consistency and convergent validity reach acceptable levels (Hair et al., 2010).

Confirmatory factor analysis showed that the fit results for the theoretically postulated five-factor model are: *χ*^2^ = 135.172, *df* = 109, *χ*^2^/*df* = 1.240, CFI = 0.996, TLI = 0.995, RMSEA = 0.018, SRMR = 0.022. The comparison of fit results between the measurement model and relevant competitive models is shown in Table 3.

**Table 3:** Fit results of measurement model and competitive models.

Model	*χ* ^2^	*df*	*χ^2^*/*df*	CFI	TLI	RMSEA	SRMR
Five-factor model	135.172	109	1.240	0.996	0.995	0.018	0.022
Four-factor model: PF and CF combined	813.431	113	7.199	0.899	0.879	0.091	0.063
Three-factor model: all three feedback types combined	1404.090	116	12.104	0.815	0.783	0.122	0.083
Single-factor model	2056.228	119	17.279	0.721	0.681	0.148	0.096

The fit of the five-factor model is superior to the three competitive models, statistically validating the proposed three-dimensional structure of teacher feedback, and supporting the differentiation of positive feedback, constructive feedback, negative feedback, self-efficacy, and intrinsic motivation at the measurement level. The Fornell–Larcker criterion test showed (Fornell and Larcker, 1981) that the square root of the AVE for each construct was higher than the absolute values of its correlation coefficients with other constructs, supporting discriminant validity.

### 4.4 Descriptive statistics, correlations, and group differences

The mean for positive feedback is 3.688, constructive feedback is 3.533, negative feedback is 2.579, self-efficacy is 3.380, and intrinsic motivation is 3.646. The absolute values of skewness for each variable do not exceed 0.466, and kurtosis absolute values do not exceed 0.476, showing no obvious univariate distributional deviations. The descriptive statistics and Pearson correlation coefficients for each variable are shown in Table 4.

**Table 4:** Descriptive statistics, correlation coefficients, and discriminant validity.

Variable	*M*	*SD*	1	2	3	4	5
1. PF	3.688	0.863	**0.797**				
2. CF	3.533	0.849	0.361***	**0.783**			
3. NF	2.579	0.915	−0.383***	−0.384***	**0.822**		
4. SE	3.380	0.871	0.526***	0.513***	−0.520***	**0.802**	
5. IM	3.646	0.830	0.456***	0.538***	−0.541***	0.640***	**0.790**

Bold values on the diagonal are the square roots of AVE, off-diagonal values are Pearson correlation coefficients. ***p < 0.001.

Positive feedback and constructive feedback are both positively correlated with self-efficacy and intrinsic motivation, while negative feedback is negatively correlated with both. The correlation coefficient between self-efficacy and intrinsic motivation is *r* = 0.640, and all core correlation coefficients reach *p* < 0.001.

Gender difference analysis only compared males and females; 7 participants who chose “Other or prefer not to say” were not included in this test. Differences across gender and teaching format groups for all five core variables did not reach significant levels. The independent samples *t*-test and one-way ANOVA results are detailed in Table 5.

**Table 5:** Group differences by gender and teaching format.

Variable	Male *M*	Female *M*	Gender *t*	*p*	Cohen’s *d*	Format *F*	*p*	*η* ^2^
PF	3.716	3.671	0.646	0.519	0.051	0.341	0.796	0.001
CF	3.541	3.533	0.114	0.909	0.009	0.683	0.562	0.003
NF	2.649	2.550	1.372	0.171	0.108	2.208	0.086	0.009
SE	3.389	3.370	0.281	0.779	0.022	0.544	0.652	0.002
IM	3.600	3.663	−0.974	0.330	−0.076	0.945	0.419	0.004

The absolute values of Cohen’s *d* for gender differences range from 0.009 to 0.108, and the *η*^2^ for teaching format differences range from 0.001 to 0.009. Although inter-group differences are small, relevant background variables were still included in subsequent regression models.

### 4.5 Regression relationship between teacher feedback and intrinsic motivation

After controlling for age, gender, occupation status, cumulative learning duration, teaching format, initial learning motivation, average daily practice duration, time with current teacher, and self-rated learning stage, all three types of teacher feedback showed significant statistical relationships with intrinsic motivation. Specific regression statistical results are shown in Table 6.

**Table 6:** Regression results of three types of teacher feedback on intrinsic motivation.

Predictor variable	*β*	*SE*	*t*	*p*	95% CI
Positive feedback (PF)	0.207	0.031	6.647	<0.001	[0.146, 0.268]
Constructive feedback (CF)	0.342	0.031	11.090	<0.001	[0.282, 0.403]
Negative feedback (NF)	−0.318	0.031	−10.144	<0.001	[−0.380, −0.257]

Values in the table are standardized regression coefficients. Model *R*^2^ = 0.478, adjusted *R*^2^ = 0.456.

The model containing only background variables explains 5.2% of the variance in intrinsic motivation. After adding the three types of teacher feedback, the model’s *R*2 is 0.478, and adjusted *R*^2^ is 0.456. The VIF for all three feedback variables ranges from 1.250 to 1.275, showing no obvious multicollinearity.

Positive feedback and constructive feedback have a positive statistical relationship with intrinsic motivation, whereas negative feedback has a negative statistical relationship with intrinsic motivation. H1a, H1b, and H1c are supported by the data.

### 4.6 Statistical mediating effect of self-efficacy

Based on 5,000 Bootstrap resampling results (see Table 7), the statistical indirect effects of self-efficacy between the three types of teacher feedback and intrinsic motivation all reached significant levels (95% confidence intervals all exclude 0). Specifically, positive and constructive feedback are both associated with higher self-efficacy, which in turn presents a statistical mediation path to expected higher levels of intrinsic motivation (indirect effect estimates are 0.103 and 0.098, respectively); conversely, negative feedback correlates with lower self-efficacy, further manifesting as a significant negative indirect association with intrinsic motivation (indirect effect estimate is −0.097). After incorporating self-efficacy as a mediator into the model, the direct statistical associations (path c′) of all three feedback types on intrinsic motivation remain significant, indicating that self-efficacy plays a “partial mediating” statistical role. Thus, H2a, H2b, and H2c receive full data support.

**Table 7:** Bootstrap mediating effects of self-efficacy.

Feedback type	Path *a*	Path *b*	Total effect *c*	Direct effect c′	Indirect effect *ab*	95% Bootstrap CI
PF	0.304	0.338	0.207	0.104	0.103	[0.075, 0.134]
CF	0.291	0.338	0.342	0.244	0.098	[0.070, 0.131]
NF	−0.287	0.338	−0.318	−0.221	−0.097	[−0.128, −0.070]

5,000 bootstrap resamples. When testing the mediation path for any specific feedback type, the other two types of feedback were controlled as covariates to ensure model consistency of the *b* path estimate.

### 4.7 Moderating effect of learning stage and conditional indirect effects

Standardized variables were used to construct interaction terms for testing. The results show that while the main effect of the learning stage itself is not significant (*β* = 0.014, *p* = 0.818), it has a significant moderating effect on both types of teacher feedback (Table 8). Specifically, the interaction term between positive feedback and learning stage is negative, *β* = −0.135, *p* < 0.001; the interaction term between constructive feedback and learning stage is positive, *β* = 0.113, *p* < 0.001.

**Table 8:** Interaction term testing model for learning stage.

Model term	*β*	*SE*	*t*	*p*	95% CI
PF	0.316	0.030	10.405	<0.001	[0.257, 0.376]
CF	0.285	0.030	9.432	<0.001	[0.226, 0.344]
NF	−0.286	0.031	−9.300	<0.001	[−0.346, −0.225]
Learning stage	0.014	0.061	0.231	0.818	[−0.106, 0.134]
PF × Learning stage	−0.135	0.029	−4.669	<0.001	[−0.191, −0.078]
CF × Learning stage	0.113	0.029	3.907	<0.001	[0.056, 0.170]

Integrating all the core regression statistical results mentioned above, the final empirical model path of this study is shown in Figure 2.

**Figure 2:**
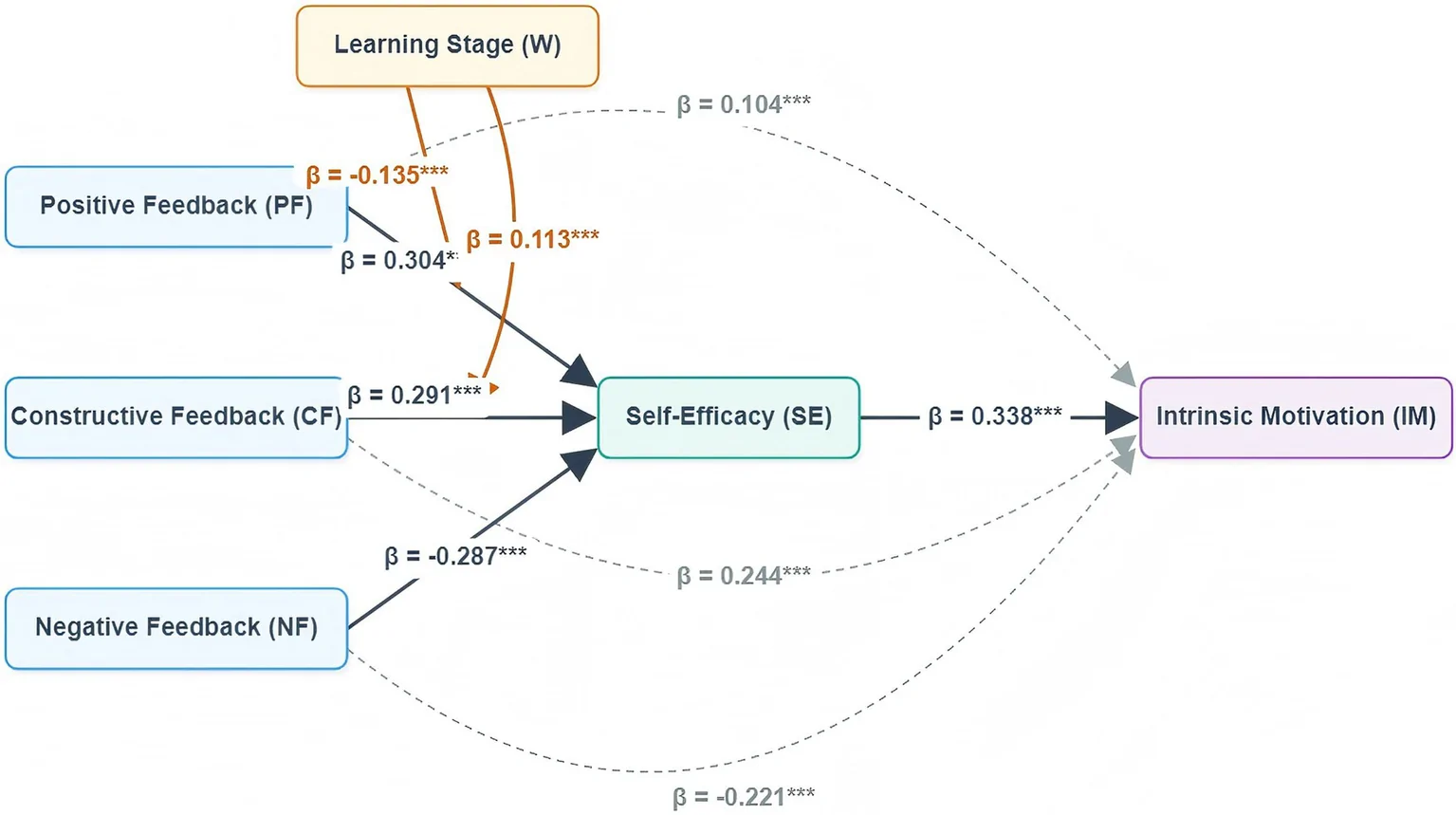
Empirical path diagram of the moderated mediation model. Solid lines represent mediation paths, dashed lines represent direct effects, orange lines represent significant moderating effects, marked values are standardized regression coefficients *β*, ***p < 0.001.

To deeply reveal the dynamic evolution of feedback effects under different learning stages, this study first extracted simple slopes for the four stage groups (Table 9).

**Table 9:** Simple slopes under different learning stages.

Feedback path	Beginner	Intermediate	Advanced	Professional
PF → SE	0.446***	0.308***	0.170***	0.032
CF → SE	0.176***	0.292***	0.408***	0.524***

***p < 0.001. The simple slope for PF in the professional group is *p* = 0.630. Regarding simple slopes, the learning stage showed entirely opposite moderating trends: the positive association between positive feedback and self-efficacy progressively weakened with ascending learning stages (becoming non-significant by the professional group); whereas the positive statistical association between constructive feedback and self-efficacy steadily strengthened as the stage elevated. Thus, H3a and H3b are supported by the data. To visually present this cross-evolution of slopes, an interaction effect plot is drawn as shown in Figure 3.

**Figure 3:**
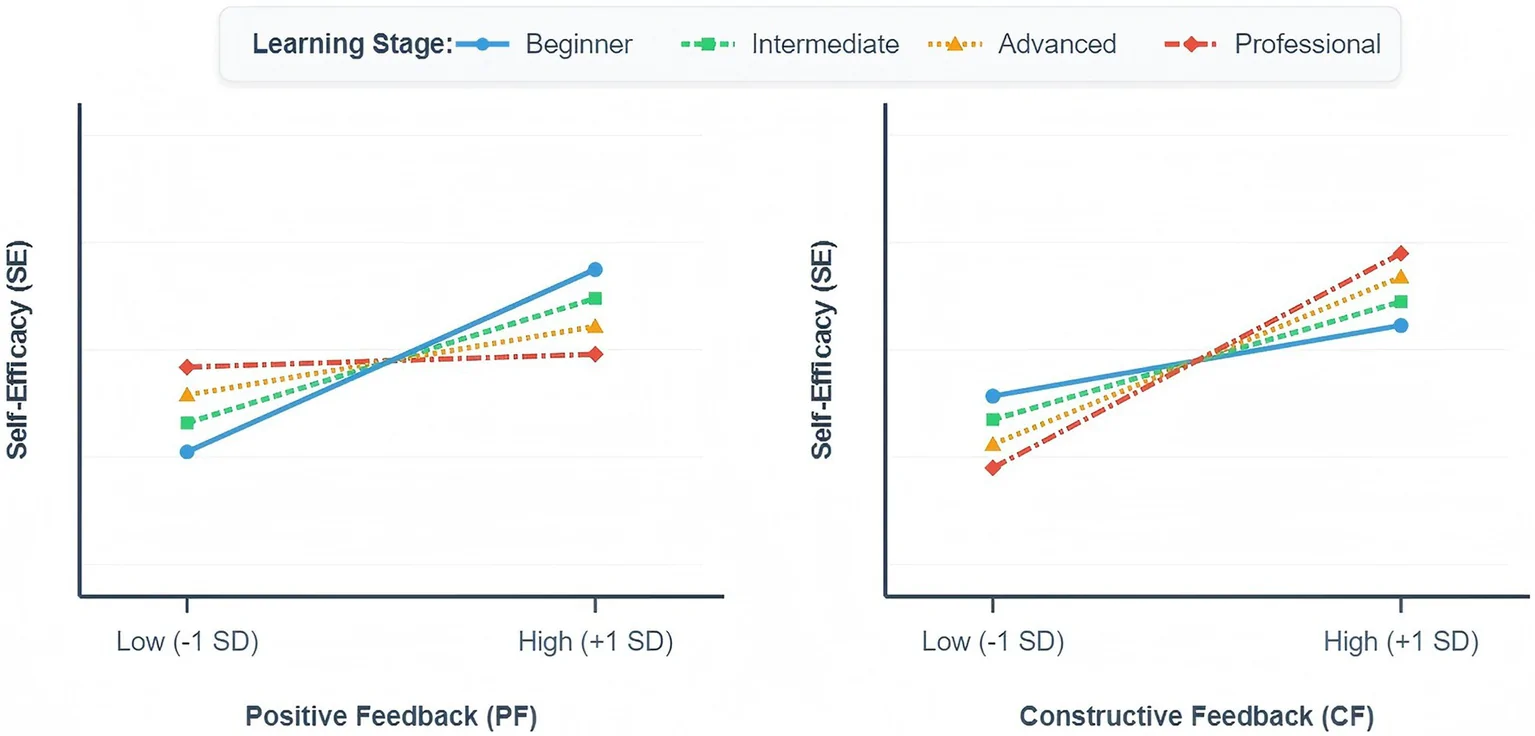
Moderating effect of learning stage between different feedback types and self-efficacy. The left Panel **(A)** shows that the strength of positive feedback positively associating with self-efficacy weakens as learning stage increases; the right Panel **(B)** shows that the strength of constructive feedback positively associating with self-efficacy strengthens as learning stage increases.

Regarding conditional indirect effects (Table 10), the strength of the conditional indirect association for positive feedback continuously weakens with increasing stages (index of moderated mediation = −0.045, 95% CI = [−0.068, −0.023]); whereas the strength of the indirect association for constructive feedback shows an expanding trend (index of moderated mediation = 0.038, 95% CI = [0.019, 0.060]). The indices of moderated mediation for both paths do not contain 0, indicating that H4 is fully supported.

**Table 10:** Conditional indirect effects.

Feedback	Stage or index	Estimate	95% Bootstrap CI
PF	Beginner	0.148	[0.108, 0.191]
PF	Intermediate	0.102	[0.074, 0.133]
PF	Advanced	0.057	[0.026, 0.091]
PF	Professional	0.011	[−0.036, 0.058]
PF	Index of moderated mediation	−0.045	[−0.068, −0.023]
CF	Beginner	0.059	[0.028, 0.091]
CF	Intermediate	0.097	[0.069, 0.130]
CF	Advanced	0.136	[0.098, 0.182]
CF	Professional	0.174	[0.121, 0.240]
CF	Index of moderated mediation	0.038	[0.019, 0.060]

The dynamic deduction results of the aforementioned indirect effect levels and confidence boundaries are visually presented via a 95% CI band graph (Figure 4).

**Figure 4:**
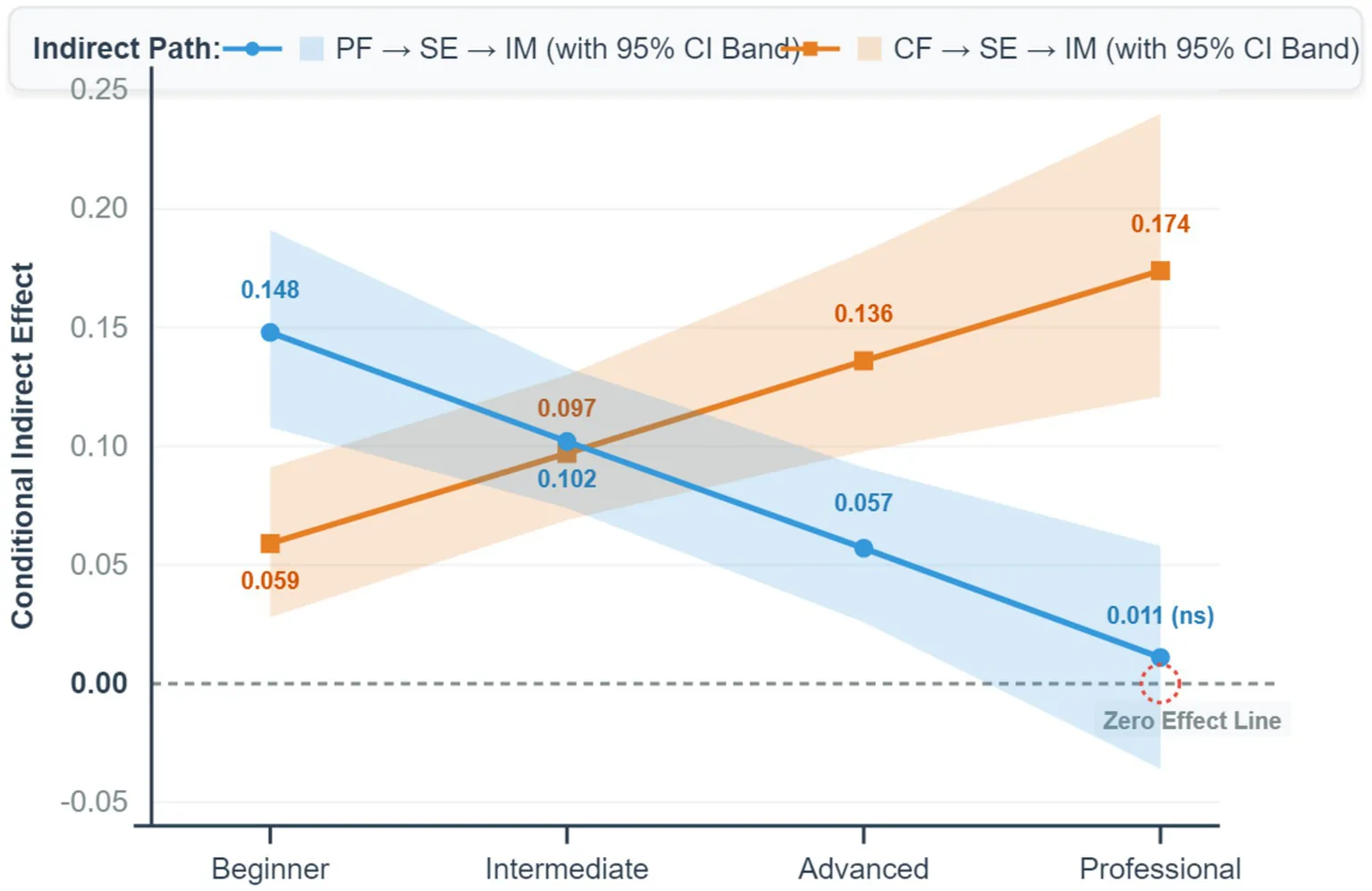
Dynamic trend band graph of conditional indirect effects corresponding to different feedback types across learning stages. Solid lines represent mediation indirect effect estimates, shaded bands represent corresponding 95% Bootstrap confidence intervals. When the dashed shaded band crosses or covers the *y* = 0 baseline level, it indicates that the conditional indirect effect is not significant.

To exclude measurement assumption biases introduced by transforming ordinal variables into continuous ones, this study conducted robustness checks on the aforementioned model trajectories. The Kruskal-Wallis H test confirmed no statistical deviations in baseline distributions across stage samples for positive feedback (*H* = 2.37, *p* = 0.499), constructive feedback (*H* = 1.07, *p* = 0.784), and self-efficacy (*H* = 1.47, *p* = 0.689). Removing the equidistant assumption and applying dummy categorical regressions, the core interaction outputs (e.g., “Professional × Constructive Feedback” *β* = 0.219, *p* = 0.0244; “Advanced × Positive Feedback” *β* = −0.261, *p* < 0.001) were highly overlapped with the statistical trajectory of the continuous model, validating the statistical robustness of the cross-group evolutionary characteristics of current moderation.

### 4.8 Qualitative text analysis of open-ended feedback

A total of 743 responses were obtained from the open-ended question, among which 207 were non-substantive narratives such as “none,” “no,” “cannot remember,” accounting for 27.86%; the remaining 536 contained specific feedback incidents. Following the systematic thematic coding of teacher feedback content, there were 279 constructive feedback incidents (37.55%), 154 negative feedback incidents (20.73%), and 103 positive feedback incidents (13.86%). The specific incident classification and percentage statistics of open-ended responses are shown in Table 11.

**Table 11:** Categorization of feedback incidents in open-ended responses.

Feedback incident category	*n*	Percentage
Constructive feedback incidents	279	37.55%
Positive feedback incidents	103	13.86%
Negative feedback incidents	154	20.73%
Non-substantive narratives	207	27.86%
Total	743	100.00%

The main feedback incident categories are mutually exclusive (i.e., each valid response is assigned to a single dominant category based on its core characteristics for statistical purposes). However, in specific analyses, the secondary psychological experience themes (such as “shame” and “ability doubt”) described by respondents are not mutually exclusive; responses within the same main category may simultaneously contain multiple psychological experience characteristics.

#### 4.8.1 Negative feedback, ability doubt, and learning avoidance

Talent labels, comparative evaluations, emotional denial, and criticism lacking improvement information frequently appeared in negative feedback texts. “Shame or self-esteem frustration” appeared 64 times, “ability doubt” appeared 51 times, and “intention to drop out or avoid” appeared 91 times.

One respondent wrote:

“Once I went to class without having effectively practiced, and was coldly scolded as being unteachable dead wood. It was so hurtful, I still remember that feeling of shame.” (P002).

Another respondent mentioned:

“There was always one note I could not play in tune, and he just kept nagging that I wasn’t as good as other students, telling me to go back and reflect. I felt extreme despair inside, doubting if there was something wrong with my IQ.” (P632).

Expressions like “unteachable dead wood,” “not as good as other students,” and “doubting my IQ” connect specific playing problems to learners’ judgments of their overall abilities. Accompanying experiences included shame, self-doubt, intentions to drop out, and reduced practice.

#### 4.8.2 Constructive feedback, cognitive clarification, and practice confidence

Constructive feedback texts concentrated on movement demonstration, technical breakdown, explanation of problem causes, and suggestions for practice steps. “Epiphany or cognitive clarification” appeared 130 times, “confidence restored” appeared 84 times, and “practice enthusiasm restored” appeared 86 times.

One respondent wrote:

“I just couldn’t learn vibrato, and the teacher picked up a pen and drew a diagram for me on the score. Instantly, a huge weight was lifted off my mind.” (P053).

Another respondent wrote:

“When I got stuck during daily practice once, the teacher marked the sheet music with specific visual cues to guide my playing. It was a huge help, and I found my enthusiasm for practice again.” (P578).

These texts link specific technical instructions with problem understanding, practice direction, ability judgment, and continued investment, forming a content correspondence with the statistical paths among constructive feedback, self-efficacy, and intrinsic motivation.

To visually reveal the micro-psychological experience flows and related behavioral tendencies accompanying different feedback incidents, this study drew an experience flow Sankey diagram (as shown in Figure 5) based on the coding frequency of the texts.

**Figure 5:**
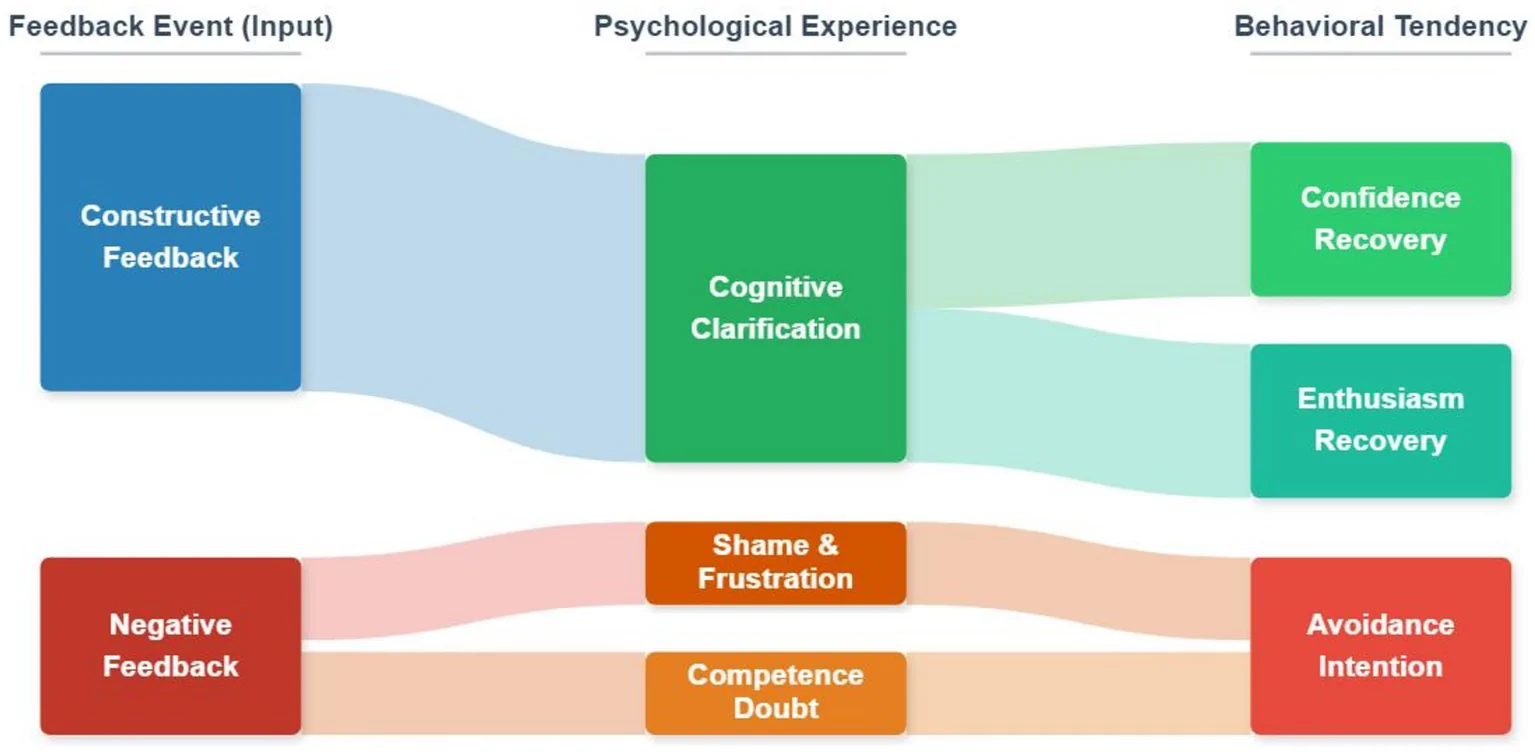
Sankey diagram of micro-experience and behavioral tendency evolution under themes analyzed from open-ended pure text. Wider node/flow heights represent higher occurrence frequencies and theme overlaps coded from open-ended questionnaire responses. Cool and warm color bands map the bidirectional development paths of positive and negative outcomes corresponding to different feedback types.

## 5 Discussion

### 5.1 Relationship between teacher feedback types and intrinsic motivation

This study distinguishes positive, constructive, and negative feedback, examining the relationships between these three types of feedback and the intrinsic motivation of adult violin learners. Results show that, after controlling for age, gender, occupation status, years of learning, teaching format, initial learning motivation, average daily practice duration, time with current teacher, and self-rated learning stage, positive and constructive feedback have positive statistical relationships with intrinsic motivation, whereas negative feedback has a negative statistical relationship with intrinsic motivation. This result supports H1a, H1b, and H1c, also indicating that teacher feedback is not a unidimensional construct; different feedback contents correspond differently to learners’ current motivational states (Brandmo and Gamlem, 2025).

The positive relationship between positive feedback and intrinsic motivation can be understood from the perspectives of ability confirmation and classroom relationship. The playing progress of adult learners is usually gradual, and some changes are not easily noticed promptly by learners. The teacher’s affirmation of effort, progress, and specific performance provides an external reference for learners, enabling them to identify changes in their intonation, bowing, or musical expression. Meanwhile, teacher recognition also conveys information of attention and acceptance, which may correspond to the competence and relatedness experiences learners feel in the classroom. According to Self-Determination Theory, these experiences are linked to learning interest, willingness to participate, and autonomous practice tendencies (Ryan and Deci, 2020; Howard et al., 2021).

The standardized regression coefficient for constructive feedback and intrinsic motivation is the highest among the three types of feedback, indicating a relatively stable positive relationship between problem identification, improvement methods, practice direction, and learners’ current motivation (Guo et al., 2025). Technical difficulties in violin learning are often specific; the same playing problem may involve movement amplitude, contact point, finger pressure, or practice sequence. Merely pointing out whether the result is correct may not help learners understand how to adjust next; when feedback includes cause explanations and actionable plans, originally general difficulties can be transformed into clearer practice tasks. For adult learners whose practice time is limited by work and family arrangements, whether feedback is actionable may be more closely tied to their sense of control over the practice process and willingness to invest (Ampler et al., 2024).

Negative feedback shows a negative relationship with intrinsic motivation. Negative feedback in the scale mainly refers to criticism lacking solutions, persistent nitpicking, and denial of talent or overall ability. Such feedback does not confine playing problems to adjustable movements or method levels, but may cause learners to interpret temporary difficulties as stable ability deficits. The competence and relatedness needs emphasized by Self-Determination Theory offer an explanation here: when classroom evaluations are linked to denial, blame, or derogatory comparisons, the interest, enjoyment, and autonomous practice willingness reported by learners are relatively low (Shriya and Bhuyan, 2025). Since this study employs a cross-sectional design, this result may also contain a reverse association; for example, learners with lower intrinsic motivation might be more likely to notice or remember a teacher’s negative expressions. Therefore, this relationship cannot be interpreted as negative feedback unidirectionally altering intrinsic motivation.

### 5.2 Statistical mediation path of self-efficacy

Self-efficacy demonstrated significant statistical indirect effects between the three types of teacher feedback and intrinsic motivation, supporting H2a, H2b, and H2c. Positive and constructive feedback form positive indirect relationships with intrinsic motivation via higher self-efficacy, whereas negative feedback forms a negative indirect statistical path with intrinsic motivation through lower self-efficacy. This result aligns with the analytical framework of “social information—ability judgment—learning state” in Social Cognitive Theory (Bandura, 1986, 1997; Miao et al., 2025).

Crucially, this mediation path elucidates the nuanced theoretical intersection between social cognitive and self-determination frameworks. The performance confirmation and actionable methods provided by positive and constructive feedback do not translate into intrinsic motivation in a vacuum; rather, they first cultivate the learner’s task-specific self-efficacy—the belief that “I can master this specific playing technique.” This concrete, domain-specific confidence subsequently manifests as the very foundation for satisfying the fundamental psychological need for competence. The talent labels or general denials in negative feedback may co-occur with lower task capability judgments, ultimately thwarting this need for competence. Expressions appearing in open-ended texts such as “doubting my IQ” and “feeling I have no talent” correspond contextually to this statistical path (Gill et al., 2026).

The three types of feedback still maintain significant direct relationships with intrinsic motivation after incorporating self-efficacy, indicating that self-efficacy only explains part of the statistical association. Teacher feedback also contains information on emotional expression, teacher-student relationships, and autonomy support, which are not directly measured by the self-efficacy scale. Teacher affirmation may be related to experiences of being respected and understood; constructive suggestions may correspond to learning autonomy and task value judgments; negative evaluations may co-occur with classroom anxiety or relational alienation. Future research could incorporate competence need satisfaction, teacher autonomy support, classroom relationships, and feedback emotional valence into the model to test if these variables can explain the remaining direct relationships (He et al., 2025).

### 5.3 Moderating patterns corresponding to learning stages

The learning stage exhibits different moderating patterns on the relationships among positive feedback, constructive feedback, and self-efficacy. The interaction term between positive feedback and learning stage is negative, while the interaction term between constructive feedback and learning stage is positive, supporting H3a and H3b. The regression relationship between the learning stage itself and self-efficacy did not reach a significant level, but the interaction terms were significant, meaning that learners at different stages may not necessarily have fixed differences in overall self-efficacy, but the relationship between feedback types and self-efficacy varies according to the learning stage.

The positive relationship between positive feedback and self-efficacy is stronger at the beginner stage and weakens as the learning stage increases. Beginners have not yet formed stable playing standards, and their judgments on intonation, movement quality, and staged progress rely heavily on teacher evaluation. The teacher’s affirmation of specific progress can serve as a reference for judging their current capabilities. Advanced and professional learners have accumulated certain practice experiences and can conduct self-evaluations based on sound outcomes, movement sensations, piece completion, and past performance; thus, the relationship between purely affirmative information and self-efficacy may be weaker (Parncutt and McPherson, 2002). In the professional group, the simple slope of positive feedback did not reach significance, which does not mean positive feedback has no classroom value, but indicates that its linear relationship with self-efficacy in this group cannot be distinguished from zero in this sample.

Constructive feedback presents an opposite pattern. Its positive relationship with self-efficacy gradually increases from the beginner to the professional stage. As learning experience accumulates, learning tasks gradually shift from basic movements and sight-reading to tone control, shifting connections, vibrato handling, phrase structure, and musical interpretation. Advanced learners generally possess more domain knowledge and are more likely to understand the technical terms and practice strategies provided by teachers. The problem diagnosis and methodological information contained in constructive feedback thus have a higher degree of correspondence with their current task needs (Zhen and Yahaya, 2026). Beginners also need specific guidance, but their ability to understand and translate complex technical information may not yet be stable, making affirmative information and basic demonstrations easier references for capability judgments.

The conditional indirect effects further demonstrate this stage difference. The indirect relationship of positive feedback through self-efficacy to intrinsic motivation weakens as the learning stage increases, with its confidence interval containing 0 in the professional group; whereas the conditional indirect relationship of constructive feedback increases as the learning stage increases. The Bootstrap confidence intervals for both indices of moderated mediation exclude 0, thus supporting H4 across both positive and constructive feedback paths. The above patterns suggest that the relationship between feedback and learners’ psychological states may depend on whether the feedback content matches their current evaluation needs and task structures.

Regarding negative feedback, the current study sets a deliberate theoretical boundary by not predefining a stage-moderated hypothesis. Existing evidence is highly conflicted on whether advanced learners possess sufficient resilience to buffer negative criticism or whether they apply stricter self-evaluative standards that make them more vulnerable to such feedback. Subsequent research could incorporate this interaction term into exploratory models and compare how learners at different stages interpret ability denials, technical criticism, and comparative evaluations.

### 5.4 Correspondence between open-ended texts and quantitative results

Open-ended responses provided a classroom context for quantitative relationships. Negative feedback texts mainly involved talent labels, comparative evaluations, criticism lacking solutions, and the resulting ability doubt, shame, and learning avoidance. Constructive feedback texts concentrated on movement demonstration, problem breakdown, cause explanation, and practice steps; learners frequently associated these with cognitive clarification, confidence restoration, and practice direction.

In negative feedback incidents, language such as “unteachable dead wood” and “not as good as other students” went beyond specific playing behaviors, extending the evaluative object from a single performance to the learner’s overall capability. Experiences occurring simultaneously with these expressions included self-doubt, intentions to quit, and reduced practice. This content resonates with the negative statistical relationships among negative feedback, self-efficacy, and intrinsic motivation. The diagrams, demonstrations, and step-by-step practices in constructive feedback incidents confined difficulties to identifiable technical links. The experiences described by learners, such as “knowing where the problem is” and “having a direction for practice,” possess conceptual links to a sense of task control and capability judgment.

The two types of data reflect correspondence and complementation rather than mutual validation. The scale results describe overall relationships at the sample level, while open texts present how participants narrate specific events (Brandmo and Gamlem, 2025). Single recollections may be influenced by current emotions, memory selection, and verbal expression styles, nor can they confirm psychological changes before and after the feedback occurred. Open-ended materials are therefore suitable for explaining classroom contexts that statistical relationships may correspond to, but are not suitable for independently confirming mediation or moderation paths.

### 5.5 Theoretical implications

The theoretical significance of this study is reflected at three levels: teacher feedback types, self-efficacy, and learning stages. When teacher feedback is differentiated into positive, constructive, and negative dimensions, each dimension exhibits different directions and strengths of relationships with intrinsic motivation. This indicates that solely using aggregate indicators like “amount of feedback” or “good/bad feedback” may obscure the differences between affirmative, methodological, and negative information.

The statistical indirect path of self-efficacy bridges Social Cognitive Theory and Self-Determination Theory. Social Cognitive Theory focuses on how learners form task capability judgments, whereas Self-Determination Theory emphasizes the links between experiences of competence, autonomy, and relatedness to intrinsic motivation (Bandura, 1997; Ryan and Deci, 2020). The research results show that part of the relationship between teacher feedback and intrinsic motivation can be described by violin learning self-efficacy, while unexplained direct relationships still exist. Rather than treating these theoretical frameworks as parallel mechanisms, this study provides empirical support for their sequential integration: task-based self-efficacy acts as the psychological vehicle that transforms environmental feedback into the satisfaction of the overarching need for competence.

The moderation results of the learning stage further demonstrate that the statistical relationships of feedback do not remain identical across all learners. Lower-stage learners correspond more strongly to the relationship between positive feedback and self-efficacy, whereas higher-stage learners correspond more strongly to the relationship between constructive feedback and self-efficacy. This result integrates learning tasks, domain experience, and feedback needs into a single analytical framework, providing highly dynamic empirical evidence for understanding stage differences in adult instrumental learning (Nash, 2025; Jiang et al., 2026).

### 5.6 Limitations and future directions

This study employed a cross-sectional, self-report questionnaire, and all core variables were reported by learners at a single point in time. Although Harman’s first principal component explained less than 40% of the variance and the multi-factor model was superior to a single-factor model, common method bias cannot be completely ruled out. Future research could combine longitudinal tracking, practice logs, teacher evaluations, and classroom observations to measure feedback experiences, self-efficacy, and intrinsic motivation at different time points to compare sequential models.

The sample was mainly concentrated on individuals aged 18–35, females, those learning for under 3 years, and beginner to intermediate learners; the representation of older age groups and advanced/professional learners was relatively limited. Although the sample covered the full proficiency spectrum from beginner to professional, providing the necessary data foundation for testing the moderating effect of “learning stage,” the relatively low proportion of high-level samples may affect the statistical power of some simple slope results (e.g., the non-significant slope for positive feedback in the professional group). Future studies could adopt stratified sampling to balance the proportions of learners across different ages, learning stages, and teaching formats (e.g., self-taught vs. one-on-one instruction) to enhance the external validity of the conclusions.

The learning stage was operationalized by a single-item self-rated level. Although this study completed robustness checks through supplementary non-parametric tests and dummy coding at the statistical stage, treating the self-rated learning stage as a continuous moderator introduces potential inherent biases. The technical progression in violin playing is profoundly non-linear; the skill acquisition and cognitive shifts required to transition between early stages differ significantly in magnitude from the leap required to reach a professional standard. Future research could further incorporate learning years, exam grades, repertoire difficulty, and objective teacher assessments to construct a multi-indicator latent variable for ‘learning stage’ and test the strict measurement equivalence across stage groups.

Additionally, while streamlining the original psychological scales successfully minimized respondent fatigue for adult learners and maintained internal consistency, future studies might deploy full-length instruments to capture the multidimensionality of these constructs more comprehensively.

Qualitative data came from one open-ended question; although the current analysis incorporated systematic high-reliability dual-coding (Cohen’s *κ*), it was ultimately constrained by the brevity of text-based survey responses. Subsequent research could select participants from different learning stages for in-depth semi-structured interviews to compare how the same feedback content is interpreted across different classroom relationships, learning goals, and technical tasks.

## 6 Conclusion and implications

### 6.1 Conclusion

Based on cross-sectional questionnaire data from 743 adult violin learners, this study investigated the relationships among teacher feedback, self-efficacy, intrinsic motivation, and learning stages. The results indicate that positive feedback and constructive feedback are positively associated with intrinsic motivation, while negative feedback shows a significant negative correlation with intrinsic motivation. Self-efficacy exhibited a significant partial mediating role between all three types of feedback and intrinsic motivation. This empirically demonstrates a structural pathway wherein the translation of environmental feedback into task-specific confidence serves as a bridging mechanism to fulfill a learner’s broader psychological need for competence, thereby sustaining intrinsic motivation.

The learning stage differentiated the relational patterns of positive and constructive feedback. The positive relationship between positive feedback and self-efficacy is stronger at lower learning stages, whereas the positive relationship between constructive feedback and self-efficacy increases as learning stages advance. The corresponding conditional indirect effects presented the same directions: the indirect relationship of positive feedback through self-efficacy to intrinsic motivation weakened as the learning stage increased, while the corresponding indirect relationship for constructive feedback increased with the learning stage.

Open-ended texts showed that negative feedback incidents often co-occurred with ability denial, shame, and learning avoidance, whereas constructive feedback incidents were more frequently associated with problem clarification, method acquisition, and practice confidence. These materials supplemented the classroom context of quantitative relationships but, given the cross-sectional design, do not constitute causal evidence.

### 6.2 Teaching implications

Teacher feedback can be adjusted according to the learning stage and task nature. For beginner learners, feedback can include affirmations of specific efforts and staged progress, while providing concise movement demonstrations and practice requirements (Arrington, 2023). Affirmative content should point to observable behaviors, such as intonation changes, rhythmic stability, or task completion, avoiding vague ability labels.

For advanced and professional learners, feedback can center more on problem diagnosis, movement mechanisms, musical structure, and practice strategies (Gill et al., 2026). Compared to generic praise, these learners may have a greater need for information that explains the source of the problem and supports autonomous adjustment. Feedback does not need to avoid errors, but evaluations should be restricted to specific performances and modifiable methods, enabling learners to formulate subsequent practice plans accordingly.

Denial, comparisons, and talent labels in negative feedback co-occur with lower self-efficacy and intrinsic motivation. When pointing out problems, teachers can distinguish among “current performance,” “practice methods,” and “overall ability,” and provide actionable directions for adjustment. For adult learners, the technical content, expression method, and time cost of feedback are all worth considering; precise, specific feedback matched with the current task better aligns with their learning needs given limited practice time (Wieser et al., 2024).

Overall, there is a stable statistical relationship between teacher feedback and the capability judgments and intrinsic motivation of adult violin learners, and this relationship changes with feedback types and learning stages. What needs attention in teaching practice is not the quantity of feedback itself, but what information the feedback contains, what tasks it points to, and whether learners possess the experiential foundation to understand and use this information (Földi et al., 2024). By illuminating how feedback processing structurally evolves across skill development, these findings offer evidence-based insights for refining adult music pedagogy and fostering lifelong self-regulated learning.

## Data Availability

The original contributions presented in the study are included in the article/Supplementary material, further inquiries can be directed to the corresponding author.
