# Supplementary material for: A study on the relationship between teacher feedback types, self-efficacy, and intrinsic motivation among violin learners
Source: Front Psychol. 2026 Sep 30;17:1985014. doi: 10.3389/fpsyg.2026.1985014 (PMC13661580; doi:10.3389/fpsyg.2026.1985014)
Supplement: Supplementary file 1 [file Data_Sheet_1.ZIP › Supplementary Material.docx]

**Appendix A: Measurement Instruments (调查问卷题项及计分说明)**

**Instruction for participants (指导语):**
Participants were asked to rate their agreement with the following statements based on their actual experiences in violin learning. All scale items (Part II) were measured on a 5-point Likert scale ranging from 1 (*Strongly Disagree / 完全不符合*) to 5 (*Strongly Agree / 完全符合*).
*(所有量表题项均采用5点李克特量表计分：1=完全不符合，5=完全符合)*

### Part I: Demographic and Background Information (基本信息变量)

*The following items were used as control variables in the analyses.*

1.Age (年龄):

1 = 18-25 years old (18-25岁，通常涵盖大学生及初入职场青年)

2 = 26-35 years old (26-35岁)

3 = 36-45 years old (36-45岁)

4 = 46-55 years old (46-55岁)

5 = 56 years and older (56岁及以上)

2.**Gender (性别):**

1 = Male (男),

2 = Female (女)

3 = Other/Prefer not to say (其他/不愿透露)

3.**Current occupational/study status (目前职业/学习状态):**
1 = Full-time student, non-music major (全日制非音乐专业学生)
2 = Full-time student, music major (全日制音乐专业学生)
3 = Employed (在职工作者)
4 = Freelancer/Retired/Other (自由职业/退休/其他)

4.**Cumulative duration of violin learning (累计学习时长):**
1 = 1 year or less (1年及以下)

2 = 1-3 years

3 = 3-5 years

4 = 5 years or more (5年以上)

5.**Primary lesson format (主要授课形式):**
1 = Offline one-on-one (线下一对一私教)
2 = Online one-on-one (线上视频一对一)
3 = Group class (小组课/集体课)
4 = Self-taught mainly (主要靠自学)

6.**Primary motivation for initial learning (最初学习的主要原因):**
1 = Pure interest in music/violin (纯粹喜爱小提琴/音乐本身)
2 = Personal development (个人成长/提升修养需求)
3 = Emotional regulation (情绪调节/缓解压力需求)
4 = External requirements (考级/升学/他人期望等外部需求)

### **7.Average daily practice time (平均每天练琴时长):** **1 = < 30 minutes (30分钟以内)** **2 = 30-60 minutes (30-60分钟)** **3 = 1-2 hours (1-2小时)** **4 = > 2 hours (2小时以上)** **8.Duration of studying with the current teacher (跟随当前老师学习的时长):** **1 = < 6 months (不满半年)** **2 = 6-12 months (半年到1年)** **3 = 1-3 years (1-3年)** **4 = > 3 years (3年以上)** **9.Self-assessed current violin level (目前小提琴水平自我评估):** **1 = Beginner (初级/入门)** **2 = Intermediate (中级)** **3 = Advanced (高级)** **4 = Professional (专业级)**

### Part II: Psychometric Scales (心理测量量表)

#### 1. Teacher Feedback Types Scale (教师反馈类型量表)

*Constructed based on Hattie & Timperley (2007) and Koka & Hein (2003).*

**Positive Feedback (积极反馈)**

**PF1:** When I play well, my teacher explicitly praises me. (当我拉得好的时候，老师会明确地表扬我。)

**PF2:** My teacher frequently acknowledges my progress and effort in learning the violin. (老师经常肯定我在小提琴学习上的进步和努力。)

**PF3:** Even if I make only slight progress, my teacher gives me positive encouragement. (即使我只取得了微小的进步，老师也会给予我积极的鼓励。)

**Constructive Feedback (建设性反馈)**

**CF1:** When I make a mistake, my teacher not only points it out but also explains exactly how to improve (e.g., bowing or fingering adjustments). (当我出现错误时，老师不仅指出错误，还会向我解释如何具体改进。)

**CF2:** My teacher’s feedback usually focuses on “how to solve the current technical difficulty.” (老师的反馈通常集中在“如何解决当前的技术难题”上。)

**CF3:** My teacher gives inspiring advice that clarifies my direction for practice. (老师会针对我的演奏给出有启发性的建议，让我清楚练习的方向。)

**Negative Feedback (消极反馈)**

**NF1:** When I play a wrong note or rhythm, my teacher often just criticizes me without telling me what is correct. (当拉错音或节奏不对时，老师经常只是批评我，而不告诉我怎样才是对的。)

**NF2:** My teacher mostly nitpicks my playing, which makes me feel discouraged. (老师更多的时候是在挑剔我演奏中的毛病，这让我感到气馁。)

**NF3:** My teacher’s feedback sometimes makes me feel that I lack talent, rather than that my practice method is flawed. (老师的反馈有时让我觉得是因为我缺乏天赋，而不是练习方法的问题。)

#### 2. Growth Mindset Scale (成长型思维量表)

*Adapted from Dweck (2006).*

**GM1:** As long as I master the right methods and keep practicing, I can significantly improve my violin playing. (只要我掌握正确的方法并坚持练习，我就能大幅提升小提琴演奏水平。)

**GM2:** When encountering technical bottlenecks, I believe it is usually because my current practice volume or method is insufficient. (遇到技术瓶颈时，我认为这通常是因为我目前的练习量或练习方法还不够。)

**GM3:** Regardless of my current level, I can significantly change my playing ability through continuous learning. (无论我现在的水平如何，我都可以通过持续学习显著改变我的演奏能力。)

#### 3. Violin-Specific Self-Efficacy Scale (小提琴学习自我效能感量表)

*Adapted from Ritchie & Williamon (2011).*

**SE1:** Even when facing very difficult exercises or phrases, I believe I can master them by practicing scientifically. (即使遇到非常困难的单调练习或乐句，我也相信自己只要按科学方法练就能掌握它。)

**SE2:** I am confident that, under my teacher’s guidance, I can gradually overcome and correct my long-standing technical problems. (我有信心能够在老师的指导下，逐渐克服并纠正自己长期存在的演奏技术问题。)

**SE3:** If a new piece looks difficult, I believe I have the ability to gradually analyze and perform it. (如果一首新乐曲看起来很难，我也相信自己有能力逐步把它分析并演奏出来。)

**SE4:** I am sure I can manage my practice sessions effectively to achieve my current learning goals. (我确信自己能够控制好练琴的状态，达成自己现阶段设定的学习目标。)

#### 4. Intrinsic Motivation Scale (内在动机量表)

*Adapted from the Interest/Enjoyment subscale of IMI (Ryan & Deci, 2020).*

**IM1:** Practicing the violin is intrinsically interesting to me; I enjoy it. (练习小提琴对我来说本身就是一件非常有趣的事情，我乐在其中。)

**IM2:** Compared to fulfilling tasks or meeting others’ expectations, I practice mainly because I genuinely like it. (相比于应付任务或他人的期望，我练习小提琴主要是因为我自己真心喜欢它。)

**IM3:** When practicing the violin, I often feel deeply engaged and even lose track of time. (练习小提琴时，我经常会感到非常投入，甚至忽略了时间的流逝。)

**IM4:** Even without my teacher’s supervision or the pressure of graded exams, I would voluntarily practice the violin. (即使没有老师的监督或考级的压力，我也会主动去练习小提琴。)

#### 5. Continuous Learning Intention (持续学习意向量表)

*Adapted from Bonneville-Roussy & Bouffard (2015).*

**CLI1:** I am very certain that I will continue to learn and practice the violin over the next year. (在未来的一年里，我非常确定自己会继续学习和练习小提琴。)

**CLI2:** No matter how my work or life circumstances change in the future, I intend to keep the violin as a long-term hobby. (无论未来工作或生活环境发生什么变化，我都打算把小提琴作为一项长期的爱好保留下去。)

Part III: Qualitative Feedback Experience (质性反馈体验 - 供混合研究分析)
**OEQ1:** Please share a specific experience where your teacher’s feedback made you feel extremely frustrated OR extremely motivated. Please briefly describe what the teacher said and how you felt psychologically. (请分享一次您的老师让您感到极度受挫，或让您充满动力的具体反馈经历。请简述当时老师说了什么，以及您的心理感受。)
[Open-ended text box / 开放式文本框，供参与者自由填写文字]

**Scoring Guide (计分说明摘要用于数据分析):**
All scale items are directly scored to reflect their respective constructs (i.e., no reverse-coding is required). The score for each construct is calculated by taking the arithmetic mean of its corresponding items. Higher scores indicate a higher level of the respective construct. (所有量表题项均不设置反向计分，直接用于计算构念得分。各变量的最终得分由其所属题目的算术平均值计算得出。得分越高代表该构念（如消极反馈）的水平越高。)

### Appendix B: Data Codebook (数据编码本)

**Note to Reviewers and Replicators (致审稿人与验证者的说明):**
This codebook corresponds to the raw dataset provided (e.g., dataset.csv). It details the variable names, descriptions, data types, and coding values used in our statistical analyses. Missing data are coded as -99. All psychological scale items (PF, CF, NF, GM, SE, IM, CLI) were measured on a 5-point Likert scale (1 = *Strongly Disagree*, 5 = *Strongly Agree*).

#### Table B1. Demographic and Control Variables (人口统计学与控制变量)

| Variable Name (变量名) | Description (变量描述) | Type (数据类型) | Coding Scheme (赋值规则) |
| --- | --- | --- | --- |
| ID | Participant Identification | Numeric | 1, 2, 3… (Sequential integer) |
| AGE | Age Group (年龄段) | Categorical | 1 = 18-25; 2 = 26-35; 3 = 36-45; 4 = 46-55; 5 = ≥ 56 |
| GENDER | Gender (性别) | Categorical | 1 = Male; 2 = Female; 3 = Other/Prefer not to say |
| STATUS | Occupational Status (职业状态) | Categorical | 1 = Student (non-music); 2 = Student (music); 3 = Employed; 4 = Other |
| YEARS | Learning Duration (学习时长) | Ordinal | 1 = ≤ 1 year; 2 = 1-3 years; 3 = 3-5 years; 4 = > 5 years |
| FORMAT | Lesson Format (授课形式) | Categorical | 1 = Offline 1-on-1; 2 = Online 1-on-1; 3 = Group class; 4 = Self-taught |
| MOTIV | Initial Motivation (初始动机) | Categorical | 1 = Intrinsic interest; 2 = Personal growth; 3 = Emotion; 4 = Extrinsic |
| **PRAC** | **Daily Practice Time (日均练琴时长)** | Ordinal | **1 = < 30 min; 2 = 30-60 min; 3 = 1-2 hrs; 4 = > 2 hrs** |
| T_DUR | **Duration with Current Teacher (跟现任老师时长)** | Ordinal | **1 = < 6 mos; 2 = 6-12 mos; 3 = 1-3 yrs; 4 = > 3 yrs** |
| LEVEL | **Self-assessed Level (自评水平)** | Ordinal | **1 = Beginner; 2 = Intermediate; 3 = Advanced; 4 = Professional** |

#### Table B2. Psychometric Scale Items (心理量表题项)

| Variable Name (变量名) | Construct (所属维度) | Item Description (题目简述) |
| --- | --- | --- |
| PF1 - PF3 | Positive Feedback (积极反馈) | Teacher praise, acknowledgment of effort, and encouragement. (PF1 to PF3 correspond to items 10-12 in the survey). |
| CF1 - CF3 | Constructive Feedback (建设性反馈) | Guidance on technical improvement and inspiring advice. (CF1 to CF3 correspond to items 13-15). |
| NF1 - NF3 | Negative Feedback (消极反馈) | Criticism without solutions, nitpicking. (NF1 to NF3 correspond to items 16-18). |
| GM1 - GM3 | Growth Mindset (成长型思维) | Belief that ability can be improved through effort and method. (GM1 to GM3 correspond to items 19-21). |
| SE1 - SE4 | Self-Efficacy (自我效能感) | Confidence in overcoming difficulties and learning new pieces. (SE1 to SE4 correspond to items 22-25). |
| IM1 - IM4 | Intrinsic Motivation (内在动机) | Enjoyment, immersion, and autonomous practice. (IM1 to IM4 correspond to items 26-29). |
| CLI1 - CLI2 | Continuous Intention (持续意向) | Intention to keep learning and retain it as a hobby. (CLI1, CLI2 correspond to items 30-31). |
| **OEQ1** | Qualitative Data (质性反馈数据) | **Open-ended text regarding a critical feedback incident (corresponds to item 32).** |

#### Table B3. Computed Variables (计算生成的总分变量)

*The following variables were computed as the arithmetic mean of their respective items and were used in the main correlation and regression/SEM analyses.*

| Computed Variable (生成的变量名) | Formula (计算公式) | Interpretation (解释) |
| --- | --- | --- |
| PF_Mean | Mean (PF1 to PF3) | Overall perceived positive feedback |
| CF_Mean | Mean (CF1 to CF3) | Overall perceived constructive feedback |
| NF_Mean | Mean (NF1 to NF3) | Overall perceived negative feedback |
| GM_Mean | Mean (GM1 to GM3) | Level of growth mindset |
| SE_Mean | Mean (SE1 to SE4) | Level of violin-specific self-efficacy |
| IM_Mean | Mean (IM1 to IM4) | Level of intrinsic motivation |
| CLI_Mean | Mean (CLI1 to CLI2) | Level of intention to continue learning |
